# “Confined Eutectic” Strategy for Visual Refrigeration Responsive Fluorescent Materials with Easy Preparation and Multi‐Color Tunability

**DOI:** 10.1002/advs.202503779

**Published:** 2025-04-02

**Authors:** Jifang Zhao, Jiahui Du, Tianyou Qin, Sean Xiao‐An Zhang, Lan Sheng

**Affiliations:** ^1^ State Key Lab of Supramolecular Structure and Materials College of Chemistry Jilin University Changchun 130012 China; ^2^ Department of Biochemistry and Molecular Biology College of Basic Medicine Science Jilin University Changchun 130012 China

**Keywords:** confined eutectic, naked‐eye recognizable, refrigerated temperature response, solid‐solid phase transition, thermofluorochromic materials

## Abstract

The temperature response window is a very critical parameter for evaluating temperature sensing performance of thermofluorochromic materials, and its regulation has long been a key focus in this field. Especially, the indication of refrigeration temperature is of great significance as it relates to public health and food safety. However, developing fluorochromic materials capable of visually reading out refrigeration temperature remains challenges. Herein, a “confined eutectic” strategy is proposed to develop fluorochromic materials that are easy to prepare and can naked‐eye recognition of refrigeration temperature. Through supramolecular interactions between dopant and host matrix, eutectic micro‐domains are formed and their crystallization point is effectively lowered into the range of 0–10 °C. The excellent crystalline host matrix provides a “confinement” effect, and thereby enhancing the crystallinity of the eutectics, which enables precise aggregation and dispersion of surrounding fluorophores, resulting in significant color changes. The mechanism is validated through variable‐temperature fluorescence spectra, confocal laser scanning microscopy and phase diagram analysis. The obtained materials exhibit high fluorescence color contrast, and can apply to diverse dyes for multi‐mode and multi‐color readout. This work not only open up new insights for refrigeration temperature responsive fluorochromic materials, but also provides a new way for visual regulation crystallinity.

## Graphical abstract

A new strategy of “confined eutectic” is proposed to achieve fluorochromic materials that can readout refrigeration temperature with naked‐eye. It leverages changes in the crystallinity of the confined eutectics in nano/micro‐domains to regulate the aggregation and dispersion states of fluorophores. This strategy is simple for material preparation and applicable to various types of dyes, enabling multiple response modes and colors.

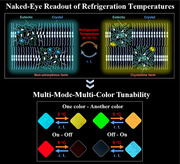

## 1 Introduction

Thermofluorochromic materials are a class of functional materials that exhibit significant changes in fluorescence color or intensity under thermal stimuli.^[^ ^1^, ^2^, ^3^, ^4^ ^]^ Since “heat” is directly related to a critical environmental parameter—temperature, these materials show great potential for applications in temperature sensing and indication.^[^ ^5^, ^6^, ^7^, ^8^ ^]^ Temperature indication technologies based on thermofluorochromic materials offer distinct advantages, including high sensitivity, multi‐scale applicability, visual recognition, and portability. As a key for evaluating the temperature sensing performance of fluorochromic materials, modulation of the temperature response window has been a major focus of research. In particularly, monitoring the refrigeration temperature (0–10 °C) is of paramount importance due to its significant impact on the quality and safety of biological products and food, as well as its essential role in safeguarding public health, optimizing resource utilization, and ensuring compliance with relevant laws and regulations.^[^ ^9^, ^10^, ^11^, ^12^ ^]^ For instance, various biological products, such as biopharmaceuticals, vaccines, and antibodies, they usually require to be stored under strict refrigeration conditions (commonly 2–8 °C) to preserve their activity and efficacy.^[^ ^11^, ^13^ ^]^ Additionally, most perishable foods need to be stored within the range of 0–10 °C^[^ ^14^, ^15^ ^]^ to extend shelf life and inhibit microbial growth.

In the past decades, although numerous thermofluorochromic materials have been developed,^[^ ^16^, ^17^, ^18^, ^19^, ^20^, ^21^, ^22^, ^23^, ^24^, ^25^, ^26^ ^]^ reports on fluorochromic materials capable of visually detecting refrigeration temperature remain scarce. Of particular note is a series of triarylboron compounds, which were skillfully designed by Yang's group.^[^ ^27^, ^28^, ^29^ ^]^ These compounds can switch between local excited (LE) and twisted intramolecular charge‐transfer (TICT) emission states in response to an exceptionally wide temperature response window (−50 to 100 °C, covering the refrigeration temperature), accompanied by continuous shifts in their fluorescence spectra. These works have inspired further thought and posed a challenge: whether it is possible to accurately identify temperature changes in narrow refrigeration temperature range using the naked eye, instead of relying on spectroscopic instruments. Although the development of related functional materials/devices holds significant theoretical and practical importance, the technical bottlenecks present considerable challenges.

In this study, we propose a new strategy of “confined eutectic” for successfully developing thermofluorochromic materials that can visually readout refrigeration temperature and are easy to fabricate. This approach enables the improvement of the crystallinity of eutectic phase change materials (PCMs) in localized nano/micro‐domains. Thus, the fluorophores doped in the confined eutectics can undergo transition between dispersion and aggregation states accompanied by significant switching in fluorescence color. Formation of the eutectics between the doped crystallization point modulator and the host matrix accurately, adjusts their crystallization point (T_C_) to the refrigeration temperature range. Simultaneously, the host matrix, with its excellent crystallinity, provides a “confining” effect that significantly enhances the crystallinity of the eutectics. In this way, it effectively addresses the technical challenge of achieving both low T_C_ and high crystallinity in conventional PCMs. This strategy can be conveniently achieved by reasonably regulating the supramolecular interactions between the host matrix, crystallization point modulator, and fluorophores. The involved mechanisms have been thoroughly elucidated. This strategy owns the advantages of simple preparation of related materials through a simple melt‐cooling process, being available for a wide range of dyes, and adjustable readout colors and modes. Additionally, this strategy involves a rare solid‐solid phase transition, which can effectively avoid the unexpected leakage risk commonly found in conventional PCMs.

## 2 Results and Discussion

Among the various thermofluorochromic systems, those based on PCMs for regulating functional fluorophores^[^ ^30^, ^31^, ^32^, ^33^, ^34^, ^35^, ^36^, ^37^, ^38^, ^39^, ^40^, ^41^, ^42^ ^]^ have garnered widespread attention, due to their simple preparation process and ability to achieve multi‐color fluorescence responses. These systems utilize the phase‐transition behavior of PCMs to modulate the state of surrounding fluorophores (e.g., aggregation or dispersion), thereby regulating the fluorescence colors. The thermal response temperatures of these systems are typically determined by the phase transition points of the PCMs (e.g., melting point, glass transition temperature, or lower critical solution temperature). However, the phase transition points of single‐component PCMs are generally above room temperature (>25 °C), which would make them impossible to respond within the refrigeration temperature range.

Recently, we have proposed a novel method^[^ ^43^, ^44^ ^]^ to construct thermofluorochromic materials by employing the melting/crystallization process of crystalline PCMs to regulate the dispersion and aggregation states of fluorophores, accompanied with fluorescence color switching. It is well known that blending two or more substances to form eutectics^[^ ^45^, ^46^, ^47^, ^48^, ^49^, ^50^ ^]^ is a common strategy to lower the melting points of materials. By adopting this approach, it is possible to reduce the melting point of crystalline PCMs into the refrigeration temperature range. However, as the melting point decreases, the crystallinity of the materials often diminishes significantly, undermining effective control over the aggregation and dispersion of fluorophores. Additionally, the PCMs melting process involved during thermally induced fluorescence color changes introduces the risk of leakage, which limits the practical applicability of such materials.

**Scheme 1:**
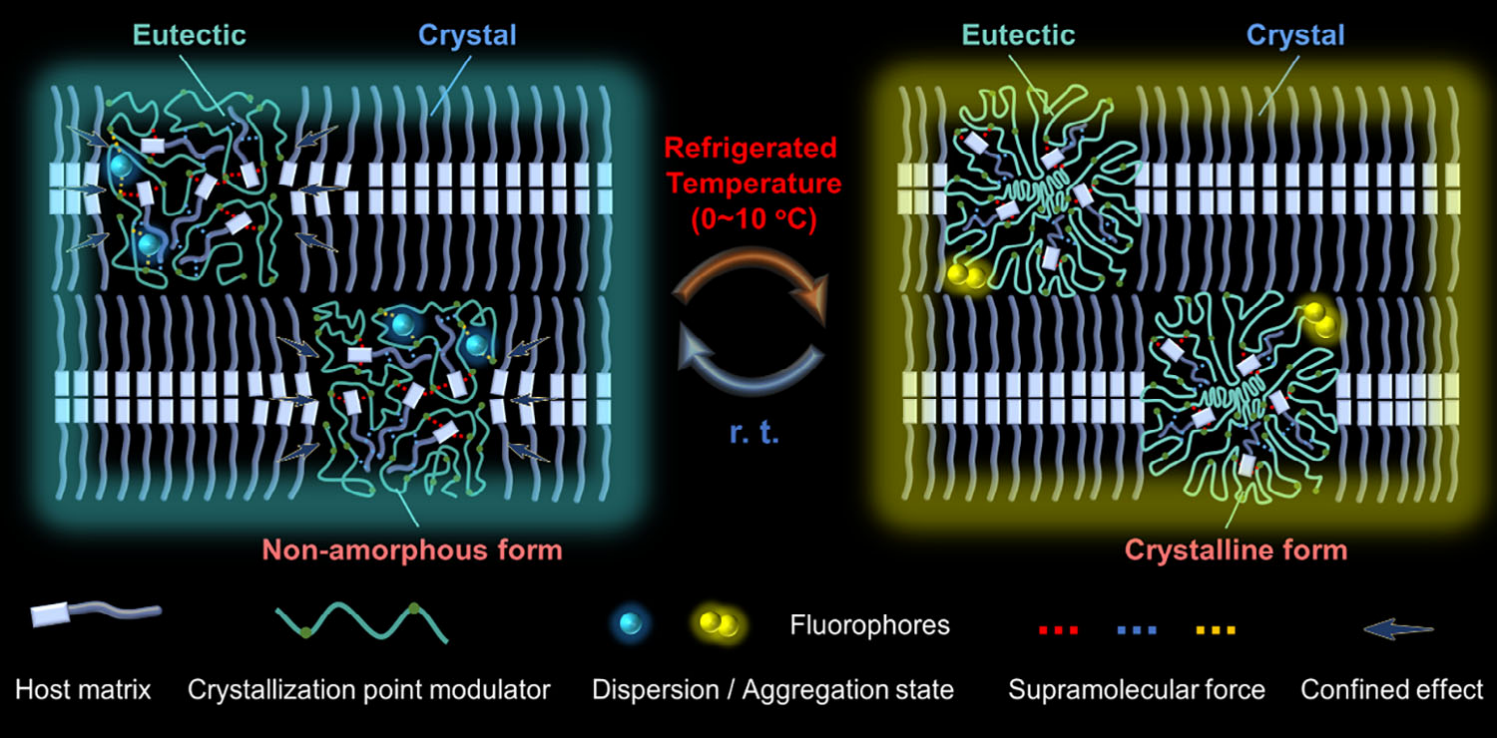
Schematic design of fluorochromic materials responding to refrigeration temperature.

Inspired by previous research and addressing the challenges mentioned above, we propose a novel “confined eutectic” strategy in this study (**Scheme** **1**). The uniqueness of this strategy is that eutectics can form in localized micro‐domains within the system by just introducing a small amount of crystallization point modulator into the host crystalline matrix, and the supramolecular interactions between crystallization point modulator and the host matrix, thereby reduce their T_C_ to the refrigeration temperature range. Simultaneously, the excellent crystallinity of the host matrix exerts a confining effect on these localized eutectics, the confining effect which can maintain eutectics at a relatively ordered state at room temperature (r. t.) and enhance its crystallinity at refrigeration temperature. This strategy successfully enables the materials to have both low T_C_ and high crystallinity simultaneously. If fluorophores with aggregative color property can be selectively distributed within these localized eutectics, it would be possible to regulate the aggregation and dispersion states (or fluorescence color) of the fluorophores by exploiting temperature‐induced crystallinity change of the confined eutectics. This approach holds promise for developing naked‐eye recognizable thermochromic fluorescence materials responding to refrigeration temperature while effectively avoiding leakage risks.

To validate this strategy, we selected dodecanoic acid (DA), a fatty acid with excellent crystallinity, as the host matrix, and coumarin‐based ester (CB) as the aggregate color‐changing fluorophore (CB exhibits yellow fluorescence in the aggregated state and cyan fluorescence in the dispersed state). In this strategy, the choice of crystallization point modulator is critical and must satisfy the following criteria: 1) It should have strong supramolecular interactions with the host matrix DA to ensure the formation of eutectics. 2) It should possess a suitable T_C_ to adjust the T_C_ of the eutectics to the refrigeration temperature range. 3) At r. t., its interactions with the fluorophores should be stronger than that between DA and fluorophores, ensuring the selective distribution of fluorophores within the localized eutectics domains. Based on these criteria, polyethylene glycol with a molecular weight of 1000 (PEG1000) was selected as the crystallization point modulator. PEG1000 has a crystallization point (T_C_: 15.2 °C) close to the refrigeration temperature range, and its molecular structure is rich in ether oxygen functional groups and long‐chain, which facilitates the formation of eutectics with DA and promote the dispersion of fluorophores such as CB.

**Figure 1:**
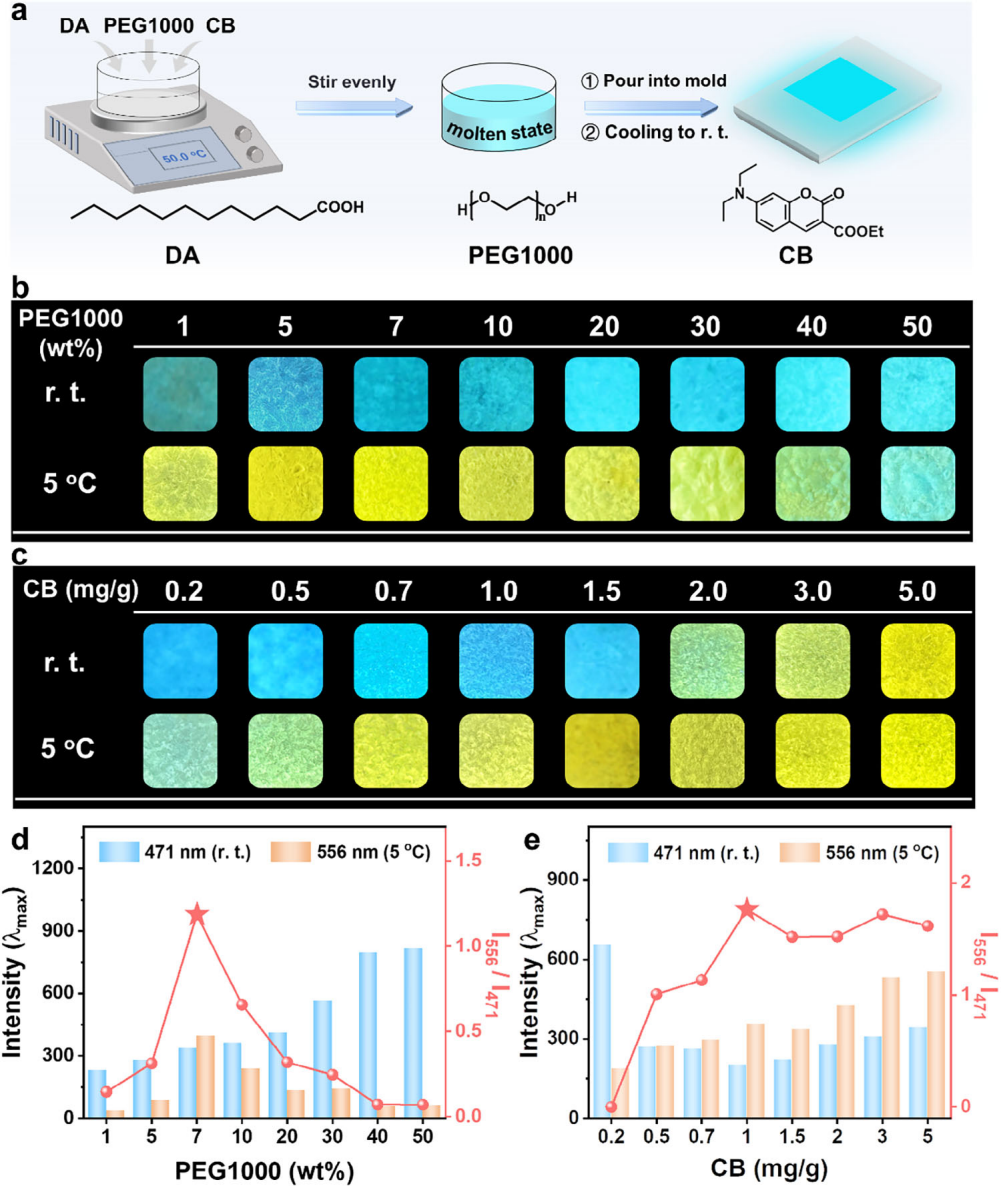
a) The preparation process of CB‐in‐DA/PEG1000; b) Fluorescence images of CB‐in‐DA/PEG1000 (*C*_CB_ = 1 mg g^−1^) with different PEG1000 concentrations at r. t. and 5 °C; c) Fluorescence images of CB‐in‐DA/PEG1000 (*ω*_PEG1000_ = 7 wt.%) with different CB concentrations at r. t. and 5 °C; d) Fluorescence intensity and fluorescence intensity ratio (I_556_ / I_471_) of CB‐in‐DA/PEG1000 (*C*_CB_ = 1 mg g^−1^) with different PEG1000 concentrations at r. t. and 5 °C; e) Fluorescence intensity and fluorescence intensity ratio (I_556_ / I_471_) of CB‐in‐DA/PEG1000 (*ω*_PEG1000_ = 7 wt.%) with different CB concentrations at r. t. and 5 °C.

First, a series of mixtures of DA, PEG1000, and CB with different ratios were prepared by heating the mixtures until molten, stirring thoroughly, and then cooling them to r. t. in molds. (**Figure** **1**a). We were excited to observe that the materials were capable of visually responding to refrigerated temperature with fluorescence switching. At r. t., the material exhibits cyan fluorescence corresponding to the dispersed state of CB, while at refrigerated temperature, it exhibits yellow fluorescence corresponding to the aggregated state of CB. As shown in Figure 1b, increasing the PEG1000 content resulted in a gradual brightening of cyan fluorescence at r. t.; however, at refrigerated temperature (e.g., 5 °C), as the PEG1000 content increases, the materials initially exhibited bright yellow fluorescence which then weakened (accompanied by partial cyan fluorescence). When the PEG1000 content exceeded 40 wt.%, the material no longer showed yellow fluorescence but displayed cyan fluorescence instead. It indicates that excessive PEG1000 hinders the aggregation of CB. Similarly, as the CB content increased (Figure 1c), the material transitioned from cyan fluorescence to yellow fluorescence at r. t. At 5 °C, the yellow fluorescence became increasingly bright with increasing CB concentrations, suggesting that higher CB levels tend to form aggregation rather than formation of the dispersed state. To determine the optimal fluorescence contrast, the fluorescence emission spectra were measured, and the intensity ratios of yellow fluorescence at 5 °C (I_556_) to cyan fluorescence at r. t. (I_471_) were plotted against PEG1000 and CB concentrations (Figure 1d,e; Figures S1 and S2, Supporting Information). The results showed that the optimal contrast was achieved when the PEG1000 content was 7 wt.% and the CB concentration was 1 mg g^−1^, as evidenced by the maximum I_556_ / I_471_ values. Consequently, a high‐contrast visual refrigeration temperature responsive fluorochromic material was successfully developed. This material was designated as CB‐in‐DA_93_PEG_7_, based on the mass percentages of PEG1000 and DA.

**Figure 2:**
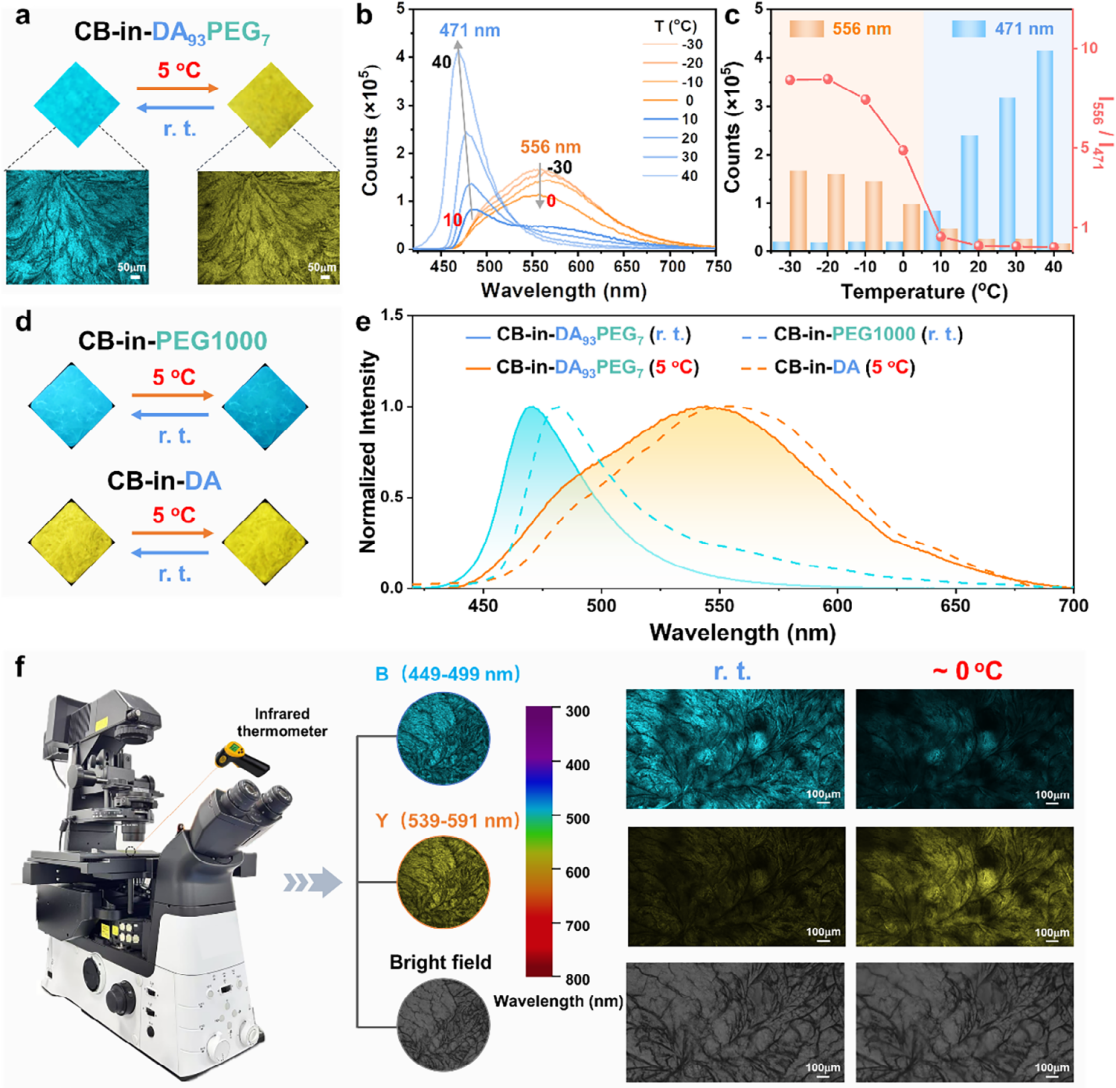
a) Fluorescence images of CB‐in‐DA_93_PEG_7_ at r. t. and refrigeration temperature (e.g., 5 °C), along with corresponding fluorescence microscope images; b) Variable temperature fluorescence spectra of CB‐in‐DA_93_PEG_7_ in the temperature range of ‐30–40 °C (at a rate of 1 °C·min^−1^); c) Plots of variable fluorescence intensity and the fluorescence intensity ratio (I_556_ / I_471_) of CB‐in‐DA_93_PEG_7_ as functions of temperature upon heating; d) Fluorescence images and e) Fluorescence spectra of CB‐in‐DA (*C*_CB_ = 1 mg g^−1^) and CB‐in‐PEG1000 (*C*_CB_ = 1 mg g^−1^) at r. t. and refrigeration temperature (e.g., 5 °C) (r. t.: slit width (3, 1.5); 5 °C: slit width (3, 3)); f) Fluorescence modes (B and Y channels) and visible light mode images of CB‐in‐DA_93_PEG_7_ at r. t. and low temperature (≈0 °C) under a laser confocal microscope; magnification: ×10. The fluorescence emission wavelength ranges for the B and Y channels are 449–499 nm and 539–591 nm, respectively.

CB‐in‐DA_93_PEG_7_ exhibits cyan fluorescence at r. t. and rapidly transitions to yellow fluorescence when placed at refrigeration temperature (e.g., 5 °C) (**Figure** **2**a). Fluorescence microscopy observations confirm that the material remained in a solid‐state morphology under both r. t. and 5 °C conditions. It is evident that the fluorescence changes observed here should be attributed to subtle variations in the molecular structure within the micro‐nano domain, occurring under a solid‐to‐solid framework. To further confirm the fluorescence transition temperature of CB‐in‐DA_93_PEG_7_, variable temperature fluorescence spectra were recorded over a wide temperature range (−30 to 40 °C) which included refrigeration temperature (Figure 2b). The results show that the material's fluorescence emission is dominated by a peak at 556 nm below 0 °C, it decreased with increasing temperature. And when the temperature is above 10 °C, a new main emission peak at 471 nm was observed. Analysis of the maximum emission intensity variation with temperature reveals that the I_556_ / I_471_ ratio underwent a significant change within the refrigeration temperature range of 0–10 °C (Figure 2c). This phenomenon clearly indicates that the fluorescence transition temperature falls within refrigeration temperature range. In addition, the fluorescence response of CB‐in‐DA_93_PEG_7_ between r. t. and 5 °C demonstrates a certain level of cyclic reversibility (Figure S3, Supporting Information).

The phenomenon in Figure 2d,e shows that the fluorescence colors and spectral of CB‐in‐DA_93_PEG_7_ at r. t. (cyan) and 5 °C (yellow) align with those of CB doped in the low‐crystallinity PEG1000 system (CB‐in‐PEG1000) and the high‐crystallinity DA system (CB‐in‐DA), respectively. Furthermore, the fluorescence characteristics of r. t. and 5 °C are comparable to those of CB in its solution state (dispersed) and solid powder state (aggregated) respectively (Figure S4, Supporting Information). These phenomena suggest that the fluorescence color change in CB‐in‐DA_93_PEG_7_ before and after refrigeration temperature stimuli would originate from the transition between the dispersion and aggregation of CB. Notably, the obvious fluorescence spectrum shift (>80 nm) of this material between r. t. and 5 °C should be attributed to the formation of excimer of CB in its aggregated state at 5 °C (Table S1, Supporting Information).

The dynamic fluorescence changes of CB in CB‐in‐DA_93_PEG_7_ during temperature variations were observed in situ using confocal laser scanning microscopy (CLSM) (Figure 2f; Figure S5, Supporting Information). With 405 nm as the excitation wavelength, the B channel (449‐499 nm) was used to capture the cyan fluorescence of CB in dispersed state, and the Y channel (539‐591 nm) was used to detect the yellow fluorescence of CB in aggregated state. Simultaneously, visible light mode was used to monitor morphology changes of the material, and a thermal imaging camera was employed for in situ temperature monitoring. At r. t., cyan fluorescence was observed across the entire field of view in the B channel, while yellow fluorescence in the Y channel was nearly absent. These phenomena indicate that CB was primarily in a dispersed state at r. t. When the temperature was reduced to refrigeration temperature (≈0 °C), the brightness of cyan fluorescence in the B channel significantly diminished, while yellow fluorescence in the Y channel intensified across the field of view. The fluorescence morphology in the Y channel at 0 °C closely mirrored the morphology of cyan fluorescence at r. t., indicating that CB transitioned from a dispersed state at r. t. to an aggregated state at 0 °C. This experiment further confirms that the fluorescence color change of CB‐in‐DA_93_PEG_7_ during the transition between r. t. and refrigeration temperature arises from the transformation between the dispersed and aggregated states of CB. In visible light mode, CB‐in‐DA_93_PEG_7_ exhibited nearly identical crystalline morphology at both r. t. and 0 °C, indicating that the crystalline structure of the host matrix remained unchanged during temperature variation. Notably, neither CB‐in‐PEG1000 nor CB‐in‐DA exhibited fluorescence color changes in response to refrigeration temperature. This demonstrates that the fluorescence color change of CB‐in‐DA_93_PEG_7_ in response to refrigeration temperature results from the synergistic interactions of DA and PEG1000 matrices.

**Figure 3:**
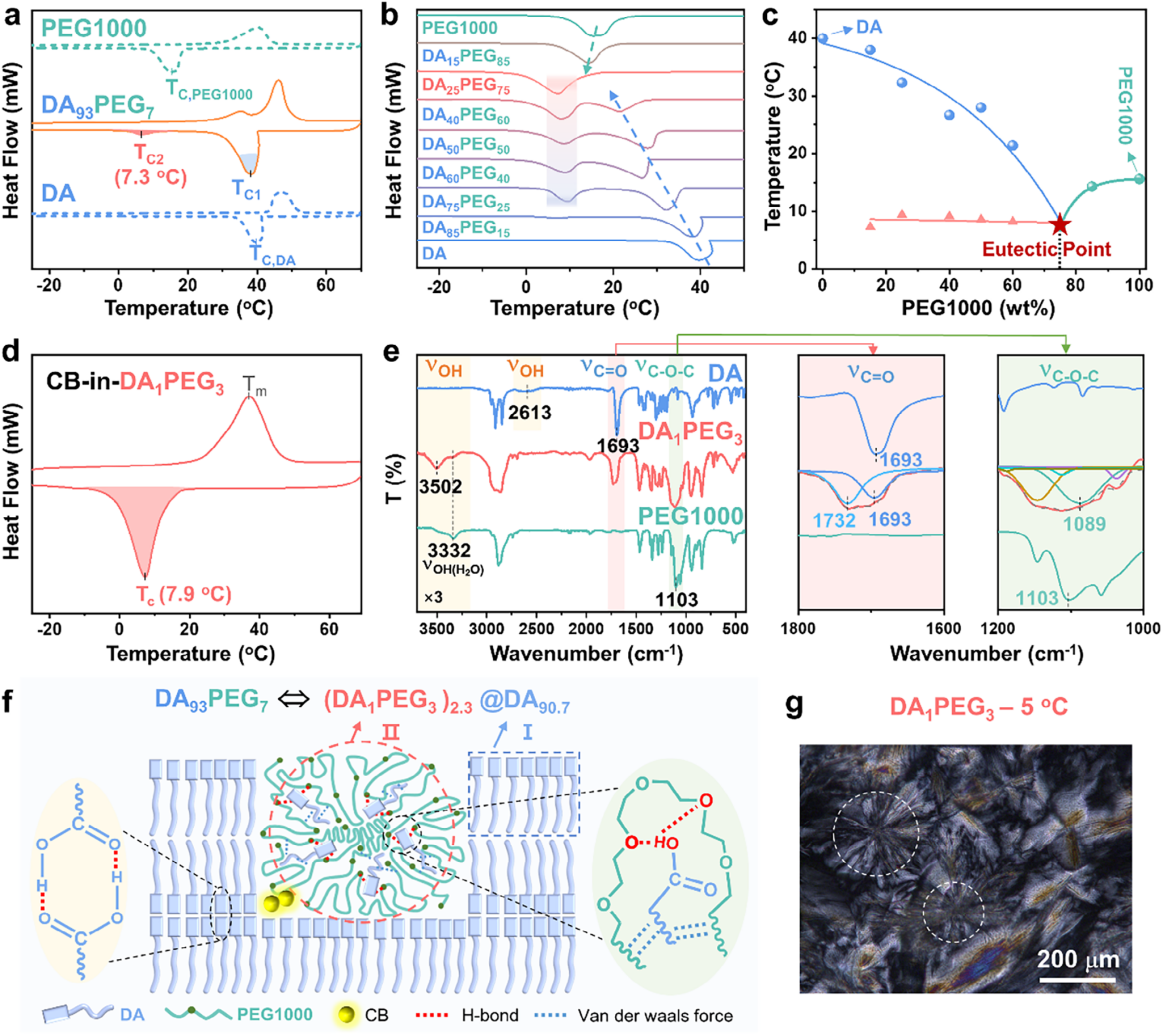
a) DSC thermograms of DA, PEG1000 and DA_93_PEG_7_; b) DSC thermograms of DA, PEG1000 and DA/PEG with different mass ratios during the cooling process (at a rate of 10 °C·min^−1^); c) Plot of the crystallization points of DA/PEG mixtures against different PEG1000 contents; d) DSC thermograms of CB‐in‐DA_1_PEG_3_ (*C*_CB_ = 1mg g^−1^); e) FT‐IR spectra of DA, PEG1000 and DA_1_PEG_3_ (orange shaded region magnified 3 times), along with the corresponding enlarged spectra for specific wavenumber ranges; f) Schematic diagram of the regional distribution of DA_93_PEG_7_; g) Polarizing microscope (POM) image of DA_1_PEG_3_.

Subsequently, the phase transition processes of heating and cooling, for the mixed matrix composed of 7 wt.% PEG1000 and 93 wt.% DA (DA_93_PEG_7_) were systematically investigated using differential scanning calorimetry (DSC), and compared with those of pure PEG1000 and DA (**Figure** **3**a). The results reveal that DA_93_PEG_7_ exhibited two distinct peaks with significantly different areas during both heating and cooling processes, indicating the presence of four‐phase transition processes in the system. Taking the cooling process as an example, a larger crystallization peak was observed with a peak temperature (T_C1_) of ≈40 °C, which closely matched the crystallization temperature (T_C_, DA) of pure DA. This suggests that this peak corresponds to the crystallization exothermic behavior of DA. Another smaller crystallization peak, with a peak temperature (T_C2_) of 7.3 °C, was significantly lower than the T_C_ of pure PEG1000 and DA, indicating that the introduction of PEG1000 as a crystallization point modulator successfully lowered the crystallization temperature of certain domains of the mixed matrix to the refrigeration temperature range (0–10 °C). Moreover, no crystallization peak of pure PEG1000 was observed during the entire cooling process, suggesting that the smaller crystallization peak (T_C2_) is attributable to the exothermic behavior of a eutectic formed between PEG1000 and DA.

To further validate this hypothesis, the cooling processes of mixtures with different mass ratios of PEG1000 and DA were analyzed. As shown in Figure 3b, with increasing PEG1000 content, all mixtures exhibited a nearly constant low‐temperature T_C_ (≈7.3 °C), which was lower than those of the pure substances, as well as a progressively decreasing T_C_ (indicated by dashed arrows) compared to DA or PEG1000. When these crystallization points were plotted against the corresponding mass fractions of PEG1000, a characteristic “V‐shaped” phase diagram was observed (Figure 3c). This diagram revealed the existence of a fixed low‐temperature T_C_ (red line). These results confirm the formation of eutectics between PEG1000 and DA. The optimal eutectic ratio was determined to be m_DA_: m_PEG1000_ = 25:75 = 1:3, denoted as DA_1_PEG_3_. During the heating process, when the melting points of these mixtures were plotted against the corresponding PEG1000 mass fractions, the optimal eutectic ratio was also found to be m_DA_: m_PEG1000_ = 1:3 (Figure S6, Supporting Information). And DA_1_PEG_3_ containing CB also had a single T_C_ (7.9 °C) during the cooling process, which closely aligns with the T_C2_ (7.3 °C) of DA_93_PEG_7_, it suggests that CB couldn't influence the T_C_ of matrix (Figure 3d). These results indicate that below the crystallization temperature, CB is indeed embedded within the DA_1_PEG_3_ eutectic structure but exists in a self‐aggregated state with relatively weak interactions with the surrounding molecules of the eutectic. Consequently, this has minimal impact on the T_C2_ properties of the original DA_1_PEG_3_ eutectic structure. Once the temperature rises above the crystallization point, however, partial reorganization and disintegration of the eutectic structure occur. The liberated free DA and PEG1000 molecules rapidly form hydrogen bonds and other supramolecular interactions with adjacent CB, transitioning it from its self‐aggregated state to a monodispersed state. Based on the enthalpy calculations for DA_93_PEG_7_ (Figure S7, Supporting Information), the phase corresponding to T_C2_ was determined to consist of ≈2.3 wt.% DA and 7 wt.% PEG1000, with a mass ratio of ≈1:3, the ratio which is consistent with the optimal eutectic ratio. These experimental results clearly indicate that the matrix of CB‐in‐DA_93_PEG_7_ is composed of two phases (or components): a large‐area phase consisting of pure DA (≈90.7 wt.%, corresponding to T_C1_) and a micro‐domain phase formed by the eutectic of PEG1000 and DA as DA_1_PEG_3_ (i.e., 2.3 wt.% DA and 7 wt.% PEG1000, corresponding to T_C2_). The crystallization phase transition behavior of the micro‐domain phase is the key factor driving the fluorescence color change of CB‐in‐DA_93_PEG_7_ in response to refrigeration temperature.

The intermolecular interactions between PEG1000 and DA in the eutectic were further investigated through infrared spectra. As shown in Figure 3e, DA exhibits no peaks above 3000 cm^−1^, except for a broad peak around 2613 cm^−1^ (blue line), indicating that stable dimers of DA have formed through strong intermolecular hydrogen bonds between the carboxyl groups (─COOH) in adjacent DA. Consequently, the hydroxyl (─OH) stretching vibration peak of free ─COOH is absent. Compared to pure DA and PEG1000, DA_1_PEG_3_ displays a new peak at 3502 cm^−1^ (pink line), which shifts to a lower wavenumber relative to the ─OH stretching vibration peak of free ─COOH in DA (typically at 3600 cm^−1^).^[^ ^51^, ^52^ ^]^ This suggests that the introduction of PEG1000 disrupts the dimeric interaction of ─COOH groups in DA molecules. Additionally, significant changes were observed in the carbonyl (─C═O) region (1800–1600 cm^−1^) and the ester group (C─O─C) region (1200–1000 cm^−1^) for DA_1_PEG_3_ compared to pure DA and PEG1000. To facilitate detailed observation, these regions were magnified, and the broad peaks of DA_1_PEG_3_ were subjected to peak deconvolution. In the ─C═O region, DA_1_PEG_3_ exhibited two distinct peaks: one aligns with the ─C═O peak of pure DA (1693 cm^−1^), which is lower than the wavenumber for free ─COOH groups, and the other appears at 1732 cm^−1^, close to the ─C═O wavenumber of free ─COOH groups. This further confirms that PEG1000 disrupts some of the hydrogen bonds between ─COOH groups in DA. In the C─O─C region, DA_1_PEG_3_ exhibits a stretching vibration peak at 1089 cm^−1^, which is shifted to a lower wavenumber compared to pure PEG1000 (1103 cm^−1^). Additionally, the ─OH peak of DA also shifts to a lower wavenumber compared to its free state. These observations indicate that the oxygen atoms (O) in PEG1000 form intermolecular hydrogen bonds with the ─OH groups in DA. Variable temperature infrared spectra further supports these findings (Figure S8, Supporting Information). As the temperature increases, the characteristic absorption peaks (e.g., C─O─C, ─OH) shift, providing additional evidence for the formation of intermolecular hydrogen bonds between PEG1000 and DA.

Based on the above experiments, the mechanism of CB‐in‐DA_93_PEG_7_ can be clarified (Figure 3f): DA, as the host matrix (accounting for 90.7 wt.% of the total matrix), forms a large crystalline region (I) due to its strong intermolecular hydrogen bonding. Meanwhile, 2.3 wt.% DA and 7 wt.% PEG1000 form eutectics (DA_1_PEG_3_) (II, referred to as a local micro‐domain), which are dispersed within the crystalline region of the host DA matrix. CB, due to its high affinity with PEG1000, is distributed within micro‐domain II. Thus, the structure of CB‐in‐DA_93_PEG_7_ can be equivalently represented as CB‐in‐(DA_1_PEG_3_)_2.3_@DA_90.7_. In micro‐domain II, including the intermolecular hydrogen bond between the ─OH groups of DA and the C─O─C groups of PEG1000, and van der Waals interactions between their alkyl chains, these two interactions cause the formation of eutectics by preferentially adsorbing DA onto the long chains of PEG1000 and make the eutectics prone to forming spherulites. Thus, these results give a reason why DA_1_PEG_3_ exhibits a “quasi‐spherulitic” crystalline morphology under polarized optical microscopy (POM) at 5 °C (i.e., circled positions) (Figure 3g; Figure S9, Supporting Information).

**Figure 4:**
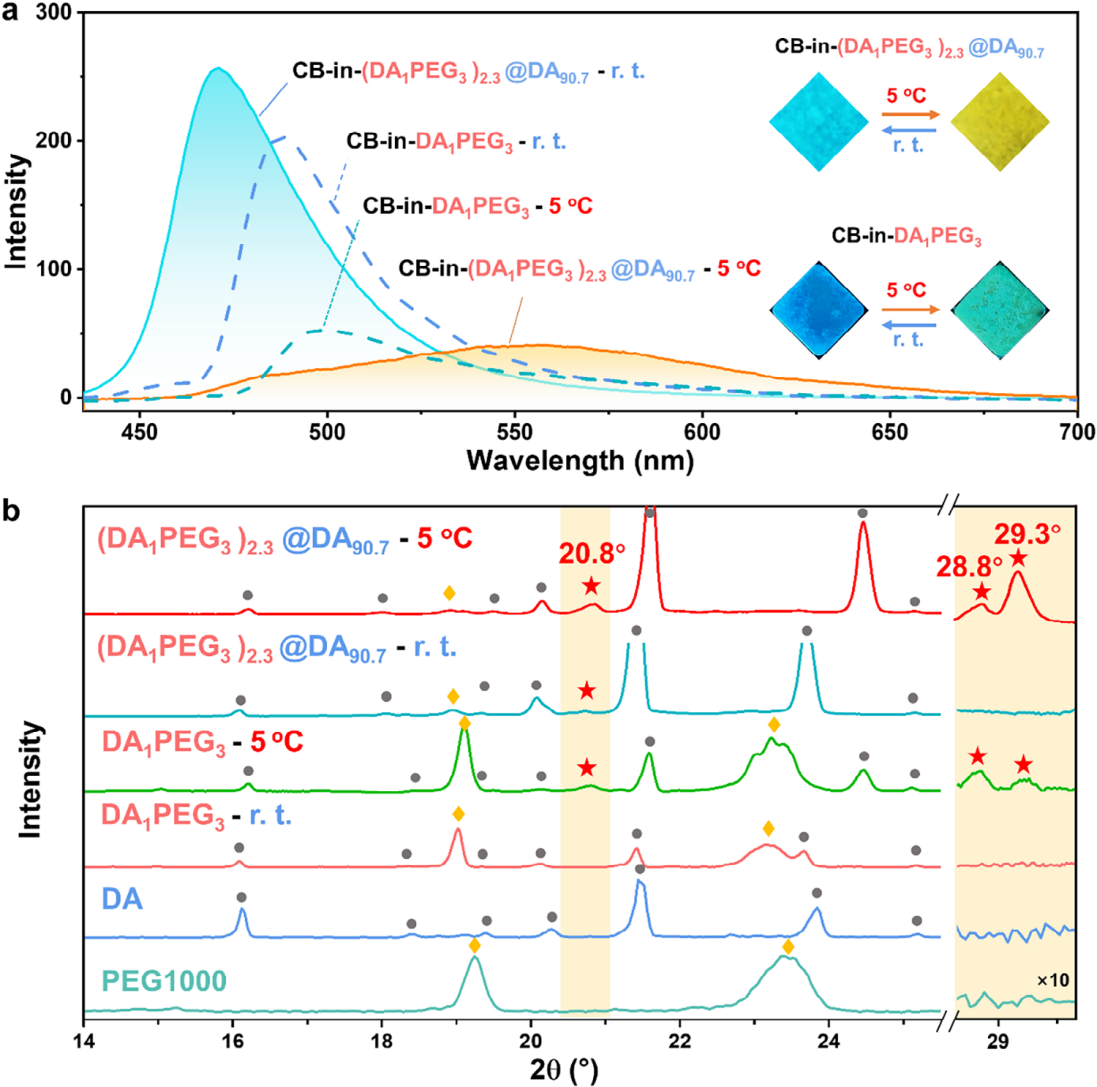
a) Fluorescence images and fluorescence spectra of CB‐in‐(DA_1_PEG_3_)_2.3_@DA_90.7_ (*C*_CB_ = 1 mg g^−1^) and CB‐in‐DA_1_PEG_3_ (*C*_CB_ ≈ 10.7 mg g^−1^) at r. t. and 5 °C (r. t.: slit width (3, 1.5); 5 °C: slit width (3, 3)); b) XRD diffraction patterns of DA, PEG1000, (DA_1_PEG_3_)_2.3_@DA_90.7_ and DA_1_PEG_3_ at r. t. and 5 °C (right side shaded region magnified 10 times) (•, ♦, ★ represent the diffraction peaks of DA, PEG1000, and the new peaks, respectively).

Next, we found that the highly crystalline host matrix DA plays a critical role in achieving high fluorescence contrast in the CB‐in‐(DA_1_PEG_3_)_2.3_@DA_90.7_ system. To simulate the color‐changing behavior of micro‐domains eutectics (DA_1_PEG_3_) within CB‐in‐(DA_1_PEG_3_)_2.3_@DA_90.7_, we estimated the concentration of CB in DA_1_PEG_3_. Due to crystallization of the host matrix DA under r. t. and the good affinity between CB and the eutectics, CB is embedded within PEG1000, resulting in a CB concentration of ≈10.7 mg g^−1^ in DA_1_PEG_3_. At this concentration, the corresponding fluorescence spectrum only shows a decrease in fluorescence intensity without significant redshift, indicating that change in the crystallinity of the eutectic (DA_1_PEG_3_) alone is insufficient for achieving aggregation of the fluorophore CB (**Figure** **4**a). This observation indirectly suggests that the host matrix DA may play a crucial role in enhancing the crystallinity of DA_1_PEG_3_ within the CB‐in‐(DA_1_PEG_3_)_2.3_@DA_90.7_ system. To further verify this hypothesis, variable temperature X‐ray diffraction (XRD) measurements were performed on the material at r. t. and 5 °C. As shown in Figure 4b, the diffraction peaks of DA_1_PEG_3_ at r. t. (DA_1_PEG_3_‐r. t.) does not show new peaks compared to pure DA and PEG1000, it only shows slight shifts for the existing peaks. However, at 5 °C (DA_1_PEG_3_‐5 °C), three new peaks are observed at 20.8°, 28.8°, and 29.3°, indicating the formation of eutectics, and these peaks are likely characteristic diffraction peaks of the eutectics (Figure S10, Supporting Information). Notably, (DA_1_PEG_3_)_2.3_@DA_90.7_ shows a diffraction peak at 20.8° even at r. t., and at 5 °C, the intensities of the peaks at 20.8°, 28.8° and 29.3° become stronger, and their widths narrower, indicating higher crystallinity of the DA_1_PEG_3_ eutectics (Figure S11, Supporting Information). This confirms that the spatial confinement effect of the host matrix DA in (DA_1_PEG_3_)_2.3_@DA_90.7_ plays a crucial role in enhancing the crystallinity of the DA_1_PEG_3_ eutectics in micro‐domains. This may be attributed to the crystallization‐confining effects of DA, which improve the structural regularity of DA_1_PEG_3_ embedded within the matrix at r. t. compared to “unconstrained” DA_1_PEG_3_ ((DA_1_PEG_3_)_2.3_@DA_90.7_‐r. t. VS. DA_1_PEG_3_‐r. t.). This structural improvement further facilitates an increase in crystallinity at refrigeration temperature.

**Figure 5:**
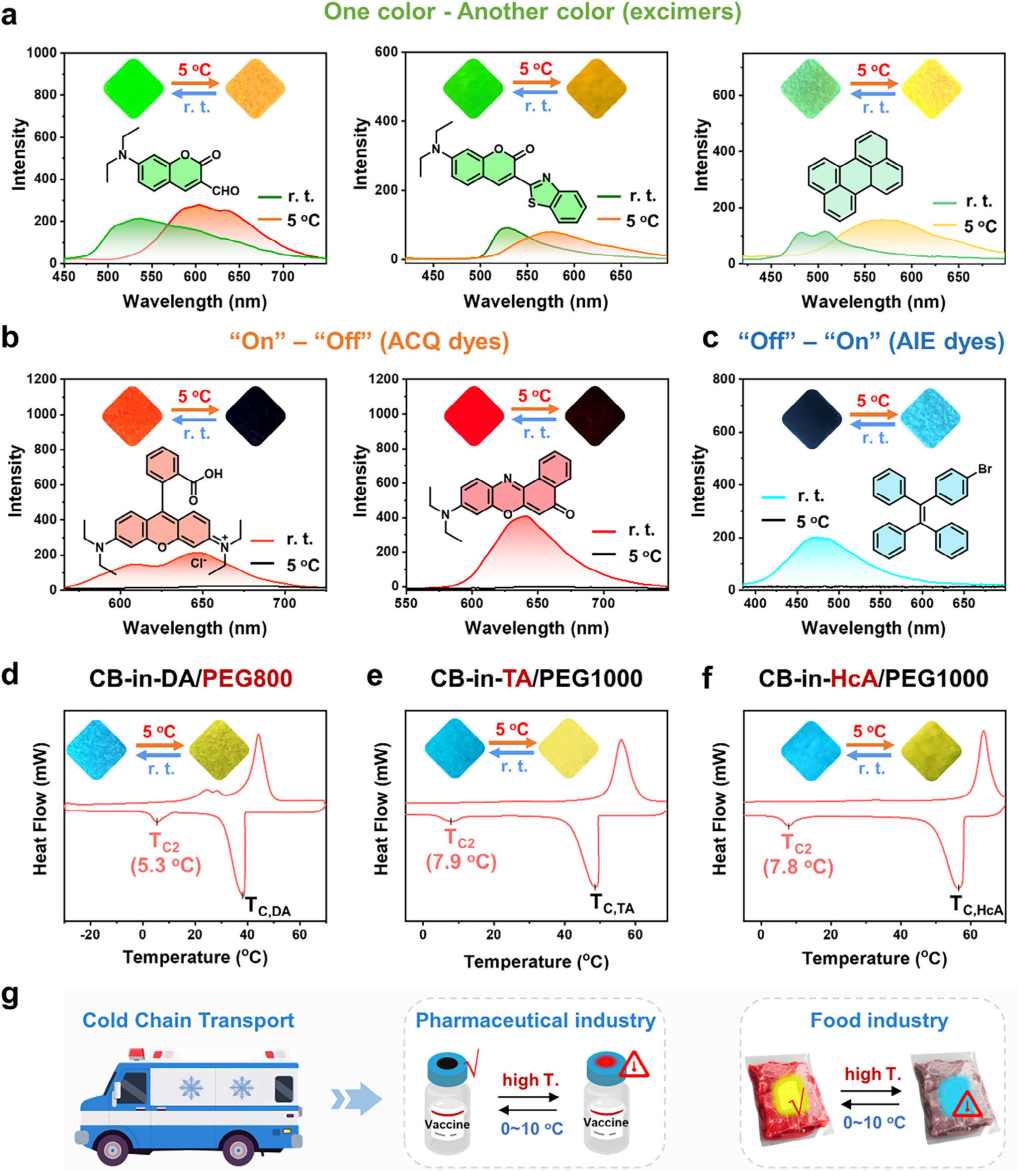
Fluorescence spectra and images of dyes‐in‐DA/PEG1000 (*ω*_PEG1000_ = 7 wt.%) with fluorescent dyes: a) coumarin derivatives, perylene; b) rhodamine B, nile red; and c) TPE‐Br; DSC thermograms, fluorescence images of d) CB‐in‐DA/PEG800 (*ω*_PEG800_ = 7 wt.%, *C*_CB_ = 1 mg g^−1^); e) CB‐in‐TA/PEG1000 (*ω*_PEG1000_ = 7 wt.%, *C*_CB_ = 1 mg g^−1^) and f) CB‐in‐HcA/PEG1000 (*ω*_PEG1000_ = 7 wt.%, *C*_CB_ = 1 mg g^−1^); g) Application prospect of refrigeration responsive fluorochromic materials.

Based on the design strategy of this work, thermofluorochromic materials with diverse fluorescence switching modes and colors can be achieved by selecting different types of aggregation‐induced fluorescence switching dyes. For example, when using excimer fluorophores (such as coumarin derivative or perylene) (**Figure** **5**a), all their fluorescence can perform transition from green or light green to orange or yellow in response to refrigeration temperature (e.g., 5 °C), accompanied by significant shifts in fluorescence emission peaks. When aggregation‐caused quenching (ACQ) fluorescent dyes are used (such as rhodamine B and nile red) (Figure 5b), these materials can undergo transition from orange‐red or red fluorescence to a non‐fluorescent state from r. t. to 5 °C, and their fluorescence dropped nearly to zero, achieving an “on‐off” fluorescence switching mode. Conversely, when aggregation induced emission (AIE) fluorescent dyes (such as TPE‐Br) are employed (Figure 5c), the materials exhibit almost no fluorescence at r. t., but emit blue fluorescence upon cooling to 5 °C, with fluorescence emission intensity increasing from zero, thus achieving an “off‐on” fluorescence switching mode. Additionally, by selecting appropriate crystallization point modulators (Figure 5d), the critical temperature range for material responsiveness to refrigeration temperature can be finely tuned. For instance, when PEG800 (T_C_: 10.9 °C) is used as a crystallization point modulator, the resulting material, CB‐in‐DA/PEG800, shows a localized eutectic micro‐domain with T_C2_ of 5.3 °C, corresponding to a critical response temperature of 5.3 °C. The associated fluorescence spectra and images demonstrate its color‐changing property in response to refrigeration temperature. Furthermore, when the host matrix is replaced with tetradecanoic acid (TA, T_C_: 50.4 °C) and hexadecanoic acid (HcA, T_C_: 59.0 °C), the resulting material, CB‐in‐TA/PEG1000 and CB‐in‐HcA/PEG1000, retain a T_C2_ of 7.9 °C and 7.8 °C, respectively. These values are essentially unchanged compared to CB‐in‐(DA_1_PEG_3_)_2.3_@DA_90.7_. The corresponding fluorescence images confirm that these materials still exhibit high fluorescence contrast in response to refrigeration temperature (Figure 5e,f). However, when crystallization point modulators with higher T_C_ values (e.g., PEG1500, PEG2000, and PEG20000) are used, the corresponding T_C2_ of the materials gradually increases. (Figures S12 and S13; Tables S2 and S3, Supporting Information). It demonstrates that the response temperature of materials achieved via the “confined eutectic” strategy primarily depends on the crystallization point modulator. Furthermore, these refrigeration responsive fluorochromic materials hold significant application potential in cold chain transport, providing precise temperature monitoring and real‐time alerts. Particularly in the pharmaceutical and food industries, these materials can help ensure the safety and quality control of products throughout the supply chain, demonstrating broad application prospects and important practical significance (Figure 5g).

## 3 Conclusion

In summary, we propose a “confined eutectic” strategy, which relies on the T_C_ regulation of PCMs to develop fluorochromic materials that can recognize refrigeration temperature (0–10 °C) by naked‐eye. The structural design and content optimization of the crystallization point modulator (PEG1000) play a critical role. By adjusting the appropriate doping mass fraction of PEG1000 and the fluorophore (CB), high‐contrast fluorescence materials can be prepared using a simple melt‐cooling process. The supramolecular interactions, including hydrogen bonding and van der Waals forces between PEG1000 and the host matrix (DA), facilitate the formation of eutectic domains within the local micro‐domains, thereby reducing their T_C_ to refrigeration temperature. Meanwhile, the excellent crystallinity of DA provides a “confinement” effect to enhance the crystallinity of the eutectic micro‐domains. This approach effectively overcomes the inherent conflict between low T_C_ and high crystallinity in conventional crystalline PCMs, enabling precise control over the aggregation/dispersion states and fluorescence color of the doped fluorophores. The formation of eutectic (DA_1_PEG_3_) between PEG1000 and DA, the associated supramolecular interactions, and the optimal eutectic mass ratio were confirmed through DSC analysis, phase diagrams, variable temperature infrared spectra, and enthalpy change calculations. For the first time, confocal laser scanning microscopy was employed to directly observe that the doped fluorophore CB undergo an in situ transition from dispersed states to aggregated states during temperature change caused by refrigeration. This transition, accompanied by the formation of excimers, results in a fluorescence color switching. Variable temperature XRD and a series of control experiments further verify that the confinement effect provided by the highly crystalline host matrix DA significantly enhances the crystallinity of the eutectic DA_1_PEG_3_ in micro‐domains.

This strategy is highly versatile and applicable to different types of fluorescence dyes, enabling various fluorescence colors and modes switching (e.g., one color to another, on‐off, and off‐on modes) for readout. Additionally, by selecting crystallization point modulator with appropriate T_C_, the critical temperature range for response to refrigeration can be finely tuned. In addition, this strategy, based on the crystallinity regulation of PCMs and involving local solid‐solid phase transitions, effectively avoids leakage risks. This work not only provides a novel strategy for the development and performance tuning of fluorochromic materials that respond to refrigeration temperature with naked eye recognizable changes, but also offers new insights into the crystallinity regulation and visual indication of polymers (e.g., PEG). Furthermore, the solid‐solid phase transitions involved not only inspire innovation in the design of smart responsive materials but also open up new avenues for PCMs to achieve efficient energy storage and release without altering their shape. This significantly expands the application potential of these materials in fields such as energy savings, display technologies, and adaptive systems.

## Conflict of Interest

The authors declare no conflict of interest.

## Supporting information



Supporting Information

## Data Availability

The data that support the findings of this study are available from the corresponding author upon reasonable request.
