# Supplementary material for: “Confined Eutectic” Strategy for Visual Refrigeration Responsive Fluorescent Materials with Easy Preparation and Multi‐Color Tunability
Source: Adv Sci (Weinh). 2025 Apr 2;12(26):2503779. doi: 10.1002/advs.202503779 (PMC12245040; doi:10.1002/advs.202503779)
Supplement: Supplementary file 1 — Supporting Information [file ADVS-12-2503779-s001.docx]

**Supplementary Information**

**"Confined Eutectic" Strategy for Visual Refrigeration Responsive Fluorescent Materials with Easy Preparation and Multi-Color Tunability**

Jifang Zhao,^1^ Jiahui Du,^1^ Tianyou Qin,^2^ Sean Xiao-An Zhang,^1^ Lan Sheng^1^*

^1^State Key Lab of Supramolecular Structure and Materials, College of Chemistry, Jilin University, Changchun 130012 (China)

^2^Department of Biochemistry and Molecular Biology, College of Basic Medicine Science, Jilin University, Changchun 130012 (China)

**Table of Contents**

1. Experimental section...............................................................................................................S-3
2. Synthesis and characterization ...............................................................................................S-4
3. Concentrations optimization of PEG1000 and CB in CB-in-DA/PEG1000 ..........................S-5
4. Reversibility of CB-in-DA_93_PEG_7_...........................................................................................S-6
5. The evidence for the formation of excimers of CB in the aggregated states.........................S-7
6. Optical path schematic diagram of confocal laser scanning microscope................................S-9
7. Phase diagram during the melting process .............................................................................S-10
8. Composition calculation of two crystallization peaks of DA_93_PEG_7_......................................S-11
9. Variable temperature infrared spectra.....................................................................................S-12
10. Polarizing microscope images of PEG1000 and DA in crystalline form..............................S-14
11. Full spectra of variable temperature XRD............................................................................S-15
12. Effect of changing crystallization point modulator and host matrix on the response temperature of thermofluorochromic materials..............................................................................................S-16
13. Supplementary Reference.....................................................................................................S-18
14. **Experimental section**

***Materials.***

Dodecanoic acid (DA) (99%), tetradecanoic acid (TA) (99%), hexadecanoic acid (HcA) (98%) were purchased from Energy Chemical (Shanghai, China). Polyethylene glycol (PEG) (Mw: 800, 1000, 1500, 2000, 20000) (known as PEG800, PEG1000, PEG1500, PEG2000, PEG20000 respectively for short) was purchased from Guangfu Fine Chemical Research Institute (Tianjin, China). Unless otherwise noted, all the other materials were purchased from Sinopharm Chemical Reagent Company Limited (Beijing, China), without further purification.

***Instruments.***

Steady state fluorescence spectra were measured using a Shimadzu RF 5301 PC spectrophotometer. Variable temperature fluorescence emission spectra were measured on an Edinburgh FLS 980 steady state spectrometer. Differential scanning calorimetry (DSC) was measured using a TA instruments Q20 under pure nitrogen gas with the heating and cooling rates both set to 10 ^o^C·min^-1^. Infrared (IR) spectra in the range of 4000 to 400 cm^-1^ were recorded using a vacuum Fourier Transform IR spectrometer VERTEX 80V-ATR. X-ray diffraction (XRD) patterns at different temperatures were measured using a German Bruker D8 Advance. Microscope images were captured in polarizing and fluorescence modes using a Leica DM4000 M microscope. Simultaneous imaging of fluorescence and visible light modes in different channels was performed using a Nikon AX confocal laser scanning microscope. The real-time temperature of the sample was measured using an infrared thermometer. Fluorescence lifetimes were measured on Edinburgh FLS 1000 using picosecond pulsed diode laser under the excitation at 400 nm.

***Test method of confocal laser scanning microscope.***

The sample, placed between a slide and a coverslip, was frozen in liquid nitrogen for 2 minutes. It was then quickly transferred to a confocal laser scanning microscope under nitrogen purge to prevent ice formation on the slide and to ensure clear optical paths for observation. Meanwhile an infrared thermometer was used to monitor the sample temperature in real time.

1. **Synthesis and Characterization**


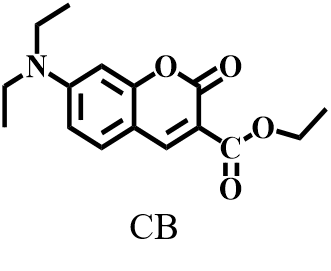
CB was synthesized according to reported literature.^[S1]^ ^1^H NMR (500 MHz, chloroform-*d*), δ: 8.42 (s, 1H), 7.36 (d, *J* = 8.9 Hz, 1H), 6.62 (dd, *J* = 8.9, 2.4 Hz, 1H), 6.47 (d, *J* = 2.3 Hz, 1H), 4.37 (q, *J* = 7.1 Hz, 2H), 3.45 (q, *J* = 7.1 Hz, 4H), 1.39 (t, *J* = 7.1 Hz, 3H), 1.23 (t, *J* = 7.1 Hz, 6H) ppm. ^13^C NMR (125 MHz, chloroform-*d*), δ: 164.39, 158.58, 158.39, 152.92, 149.30, 131.14, 109.68, 109.21, 107.88, 96.93, 61.28, 45.26, 14.51, 12.54 ppm.

1. **Concentration optimization of PEG1000 and CB in CB-in-DA/PEG1000**


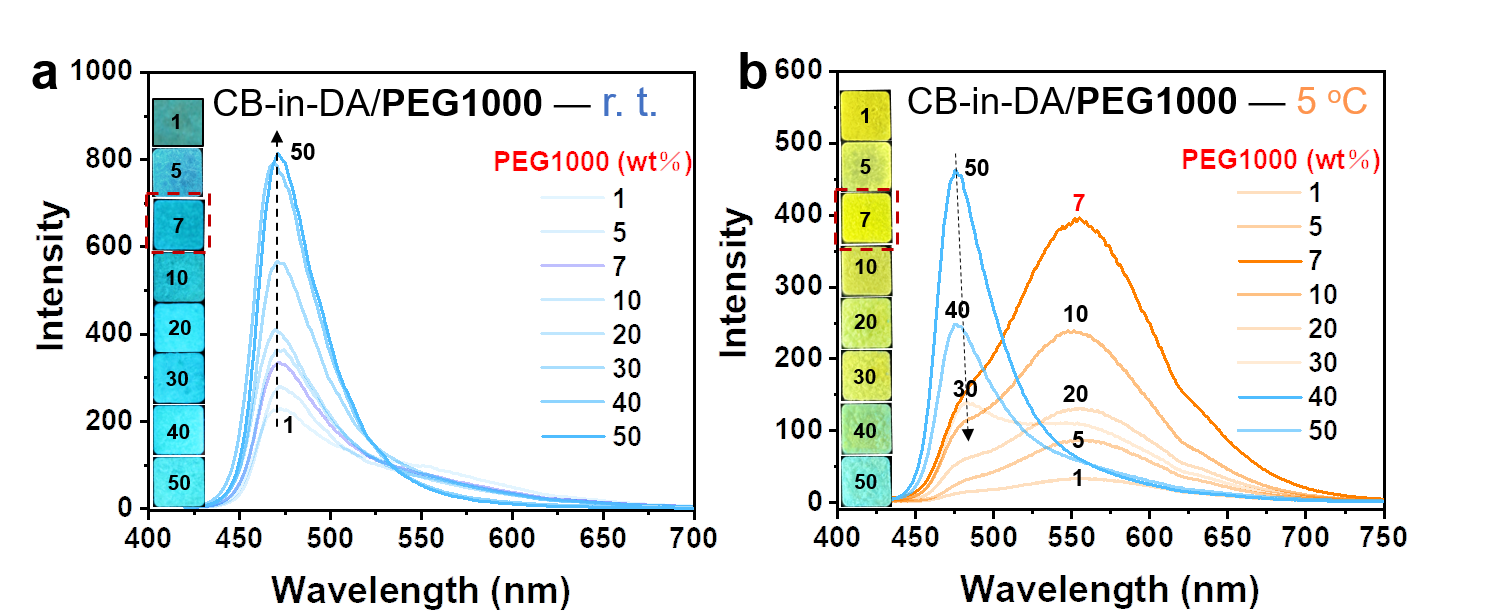


**Figure S1.** Fluorescence emission spectra and images of CB-in-DA/PEG1000 (*C*_CB_ = 1mg/g) at (a) room temperature (r. t.) (left) and (b) 5 ^o^C (right) with different concentration of PEG1000.
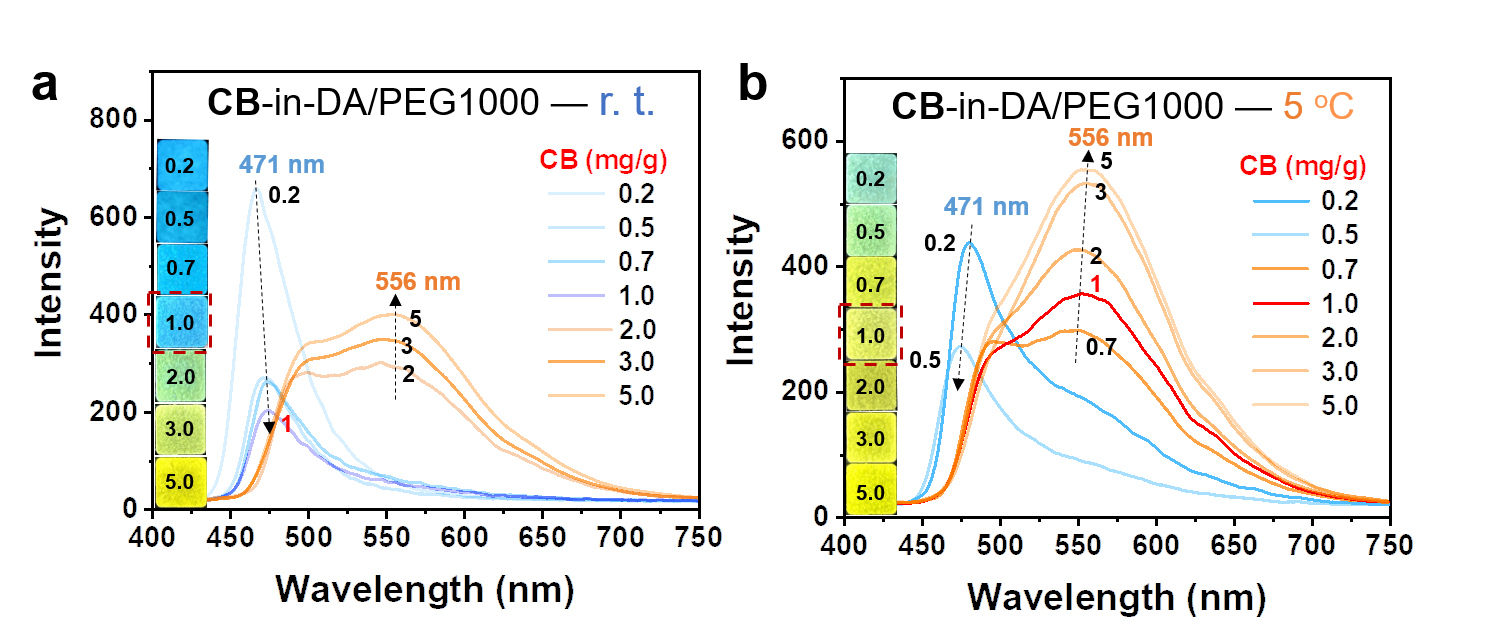


**Figure S2.** Fluorescence emission spectra and images of CB-in-DA/PEG1000 (*ω*_PEG1000_ = 7wt%) at (a) r. t. (left) and (b) 5 ^o^C (right) with different concentration of CB.

1. **Reversibility of CB-in-DA_93_PEG_7_**

*
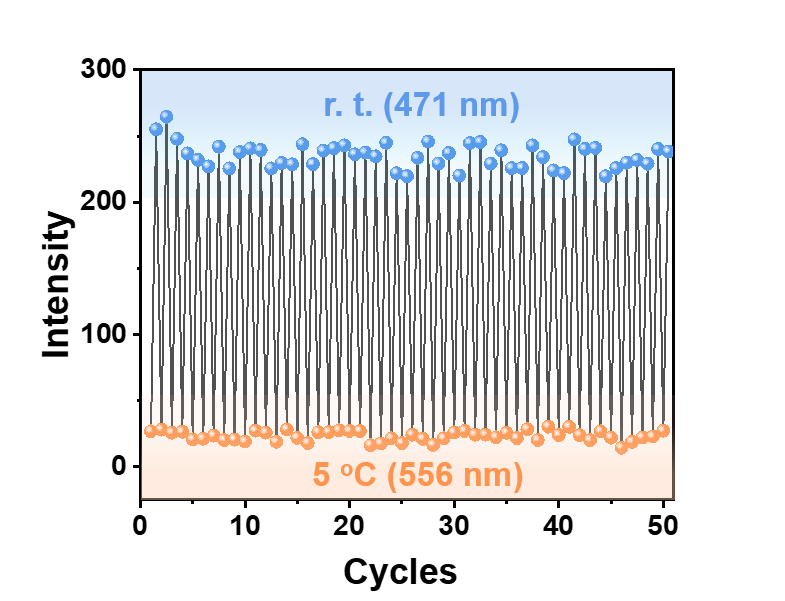
*

**Figure S3.** CB-in-DA_93_PEG_7_ reversible fluorescence response over 50 consecutive cycles upon r. t. and 5 ^o^C (r. t.: slit width (3, 1.5); 5 ^o^C: slit width (3, 3)).

1. **The evidence for the formation of excimers of CB in the aggregated states**

**
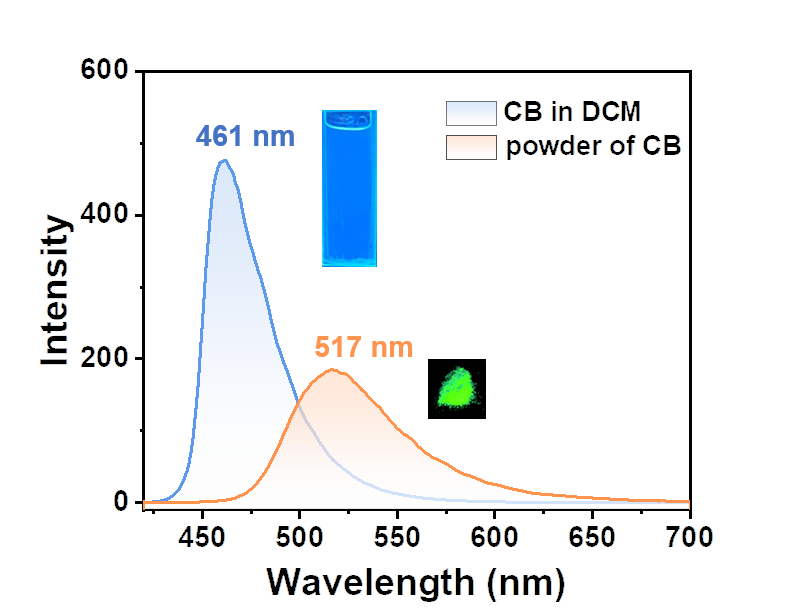
**

**Figure S4.** Fluorescence spectra and images of CB powder and its solution in dichloromethane (DCM) (*C* = 1.0 × 10^−5^ mol·L^−1^).

**Table S1.** Fluorescence lifetimes of CB-in-PEG1000 (*C*_CB_ = 1mg/g) and CB-in-DA (*C*_CB_ = 1mg/g) at r. t..

|  | τ_1_ ^[a]^ (ns) | τ_2_ ^[a]^ (ns) | τ_3_ ^[a]^ (ns) | τ_avg_ ^[b]^ (ns) | χ^2 [c]^ |
| --- | --- | --- | --- | --- | --- |
| CB-in-PEG1000 | 0.67 | 2.02 | 11.76 | 2.16 | 0.921 |
| CB-in-DA | 2.05 | 9.21 | 22.53 | 19.12 | 1.221 |

^[a]^ Fluorescence lifetime; ^[b]^ Average lifetime; ^[c]^ Goodness of fit.

The emission lifetime of CB-in-DA is longer than that of CB-in-PEG1000 (19.12 ns *VS.* 2.16 ns), which indicates that CB forms excimers in aggregation states.

1. **Optical path schematic diagram of confocal laser scanning microscope**

**
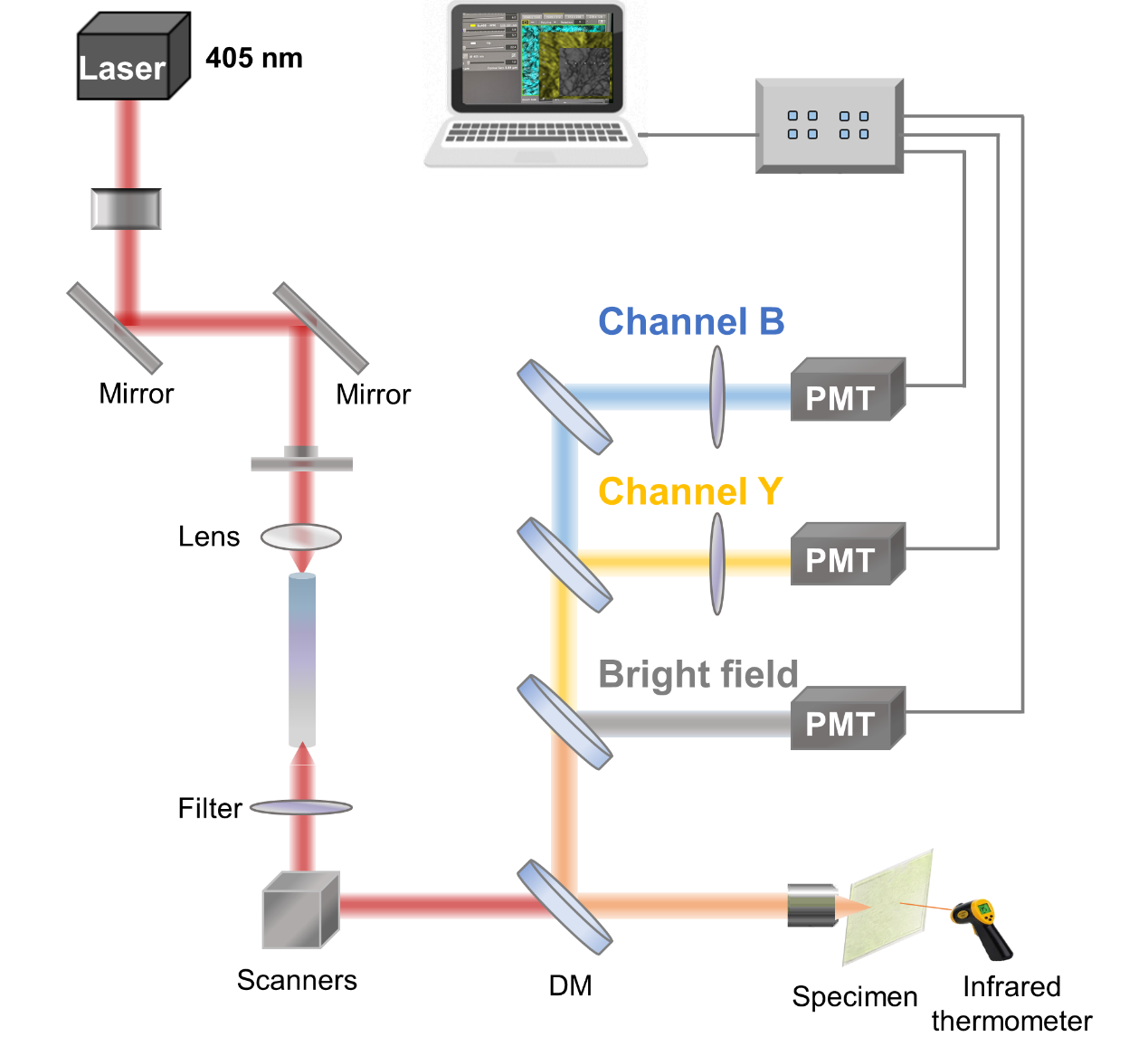
**

**Figure S5.** Schematic diagram of confocal laser scanning microscope.

1. **Phase diagram during the melting process**

**
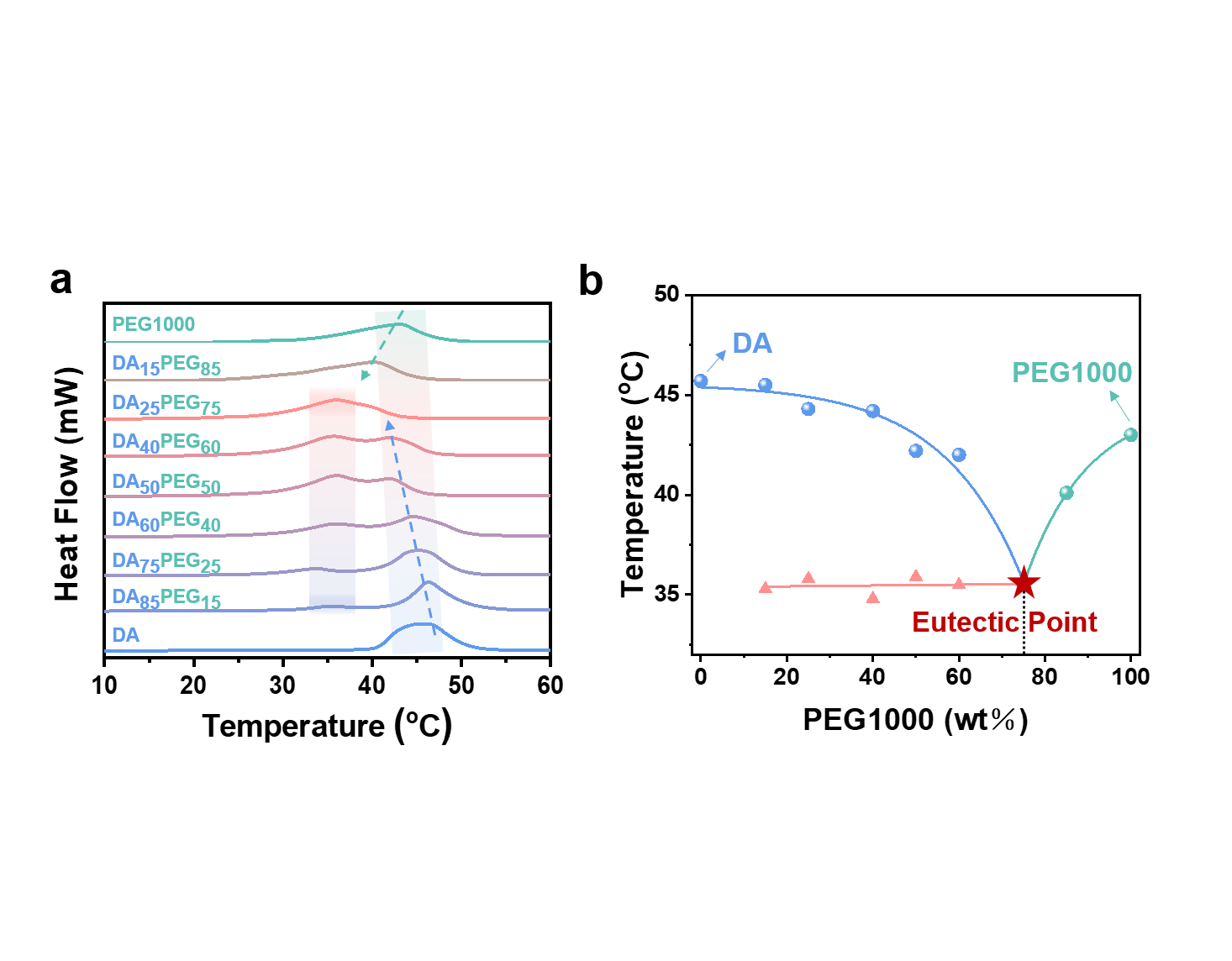
**

**Figure S6.** (a) DSC thermograms of DA, PEG1000 and DA/PEG mixtures with different mass ratios during the heating process (at a rate of 10 °C·min^-1^); (b) Plot of the melting points of DA/PEG mixtures against different PEG1000 contents.

1. **Composition calculation of two** **crystallization peaks of DA_93_PEG_7_**

**
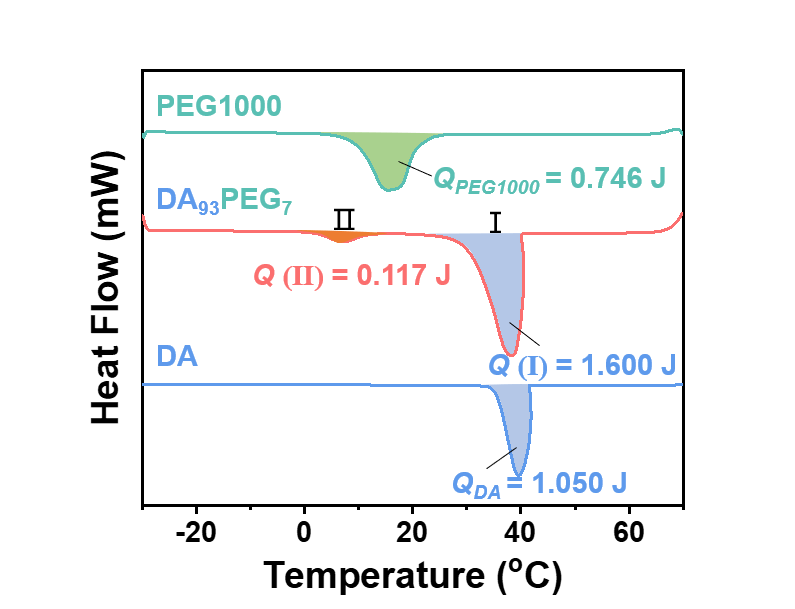
**

**Figure S7.** DSC thermograms of DA、DA_93_PEG_7_ and PEG1000 in the cooling process.

The composition of the two crystallization peaks, named region Ⅰ and region Ⅱ in DA_93_PEG_7_, was determined from following equations:


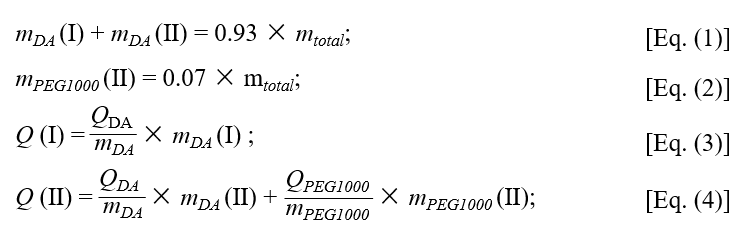


Where *m_DA_* (Ⅰ) is the mass of DA in region I of DA_93_PEG_7_; *m_DA_* (Ⅱ) and *m_PEG1000_* (Ⅱ) are the masses of DA and PEG1000 in region II of DA_93_PEG_7_; *m*_total_ is the total mass of DA_93_PEG_7_ (10.280 mg for the DA_93_PEG_7_); *Q* (Ⅰ) and *Q* (Ⅱ) are the exothermic enthalpies of regions Ⅰ and Ⅱ in DA_93_PEG_7_, respectively (1.600 J for the region Ⅰ, 0.117 J for the region Ⅱ); *Q_DA_* and *Q_PEG1000_* are the exothermic enthalpies of DA and PEG1000 respectively (1.050 J for DA, 0.746 J for PEG1000); *m*_DA_ and *m*_PEG1000_ are the masses of DA and PEG1000 respectively (6.190 mg for DA, 7.460 mg for PEG1000).

It can be obtained from the above equations: *m_DA_* (Ⅰ) ≈ 9.33 mg; *m_DA_* (Ⅱ) ≈ 0.23 mg; *m_PEG1000_* (Ⅱ) ≈ 0.7196 mg; Therefore, in region II, *m_DA_* : *m_PEG1000_* ≈ 1 : 3.That is, the composition of region I is about 90.7 wt％DA; and the composition of region II is 7wt% PEG1000 and 2.3 wt％DA.

1. **Variable temperature infrared spectra**

**
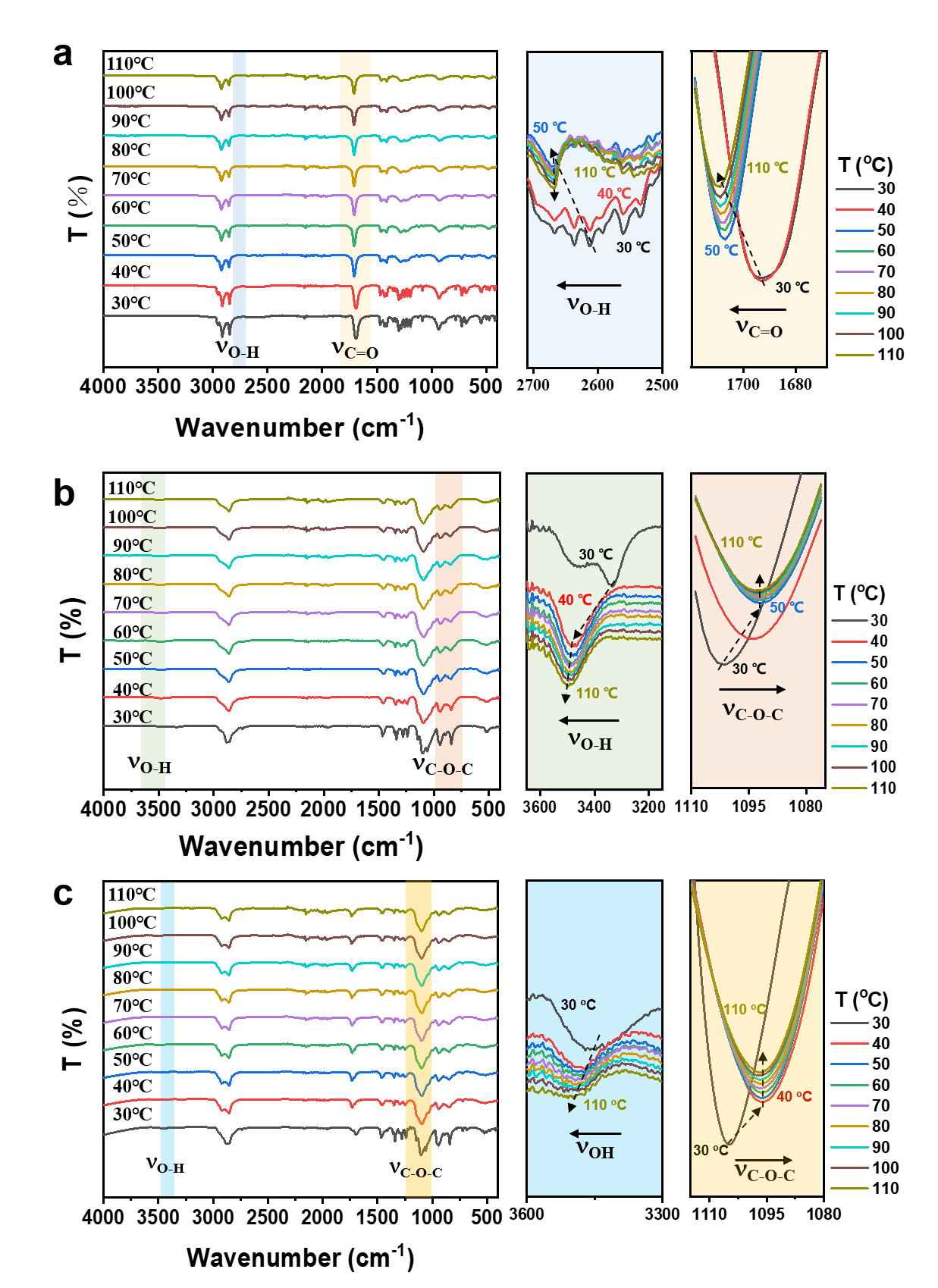
**

**Figure S8.** Infrared spectra of (a) DA, (b) PEG1000 and (c) DA_1_PEG_3_ in the heating process from 30 to 110 ^o^C.

As shown in Figure S8a, During the phase transition of DA between 30 ^o^C and 50 ^o^C, the C=O stretching vibration peak shifts to higher wavenumbers, increasing from 1693 cm⁻¹ to 1706 cm⁻¹. This trend continues up to 110 ^o^C, where the peak reaches 1708 cm⁻¹. Simultaneously, the O-H stretching vibration peak also shifts to higher wavenumbers throughout the entire heating process (30 ^o^C to 110 ^o^C), increasing from 2613 cm⁻¹ to 2669 cm⁻¹, with a narrowing of the peak width. These changes indicate that the intermolecular hydrogen bond network is gradually disrupted as the temperature rises.

As shown in Figure S8b, for PEG1000, the O-H stretching vibration peak undergoes a significant shift to higher wavenumbers during heating from 30 °C to 110 °C, increasing from 3334 cm⁻¹ to 3492 cm⁻¹. Within the phase transition range (30 ^o^C to 50 ^o^C), the C-O-C stretching vibration peak shifts to lower wavenumbers, decreasing from 1101 cm⁻¹ to 1091 cm⁻¹. This shift is attributed to the increased disorder and relaxation of the polymer chains at higher temperatures, which weakens the hydrogen bonding and lowers the vibration frequency. When the temperature reaches 110 ^o^C, the C-O-C stretching vibration peak shows minimal further change (from 1091 cm⁻¹ to 1092 cm⁻¹).

As shown in Figure S8c, for DA_1_PEG_3_, as the temperature increases from 30 ^o^C to 110 ^o^C, the O-H stretching vibration peak shifts to higher wavenumbers, increasing from 3472 cm⁻¹ to 3499 cm⁻¹. Within the phase transition temperature range (30 ^o^C to 40 ^o^C), the C-O-C stretching vibration peak shifts to lower wavenumbers, decreasing from 1105 cm⁻¹ to 1095 cm⁻¹. Further heating to 110 ^o^C results in negligible additional change in the C-O-C stretching vibration peak (1095 cm⁻¹ vs. 1096 cm⁻¹). These observations suggest that the intermolecular hydrogen bonds between the oxygen atoms in PEG1000 and the -OH groups of DA are progressively disrupted as the temperature increases.

1. **Polarizing microscope** **images of PEG1000 and DA** **in crystalline form**

**
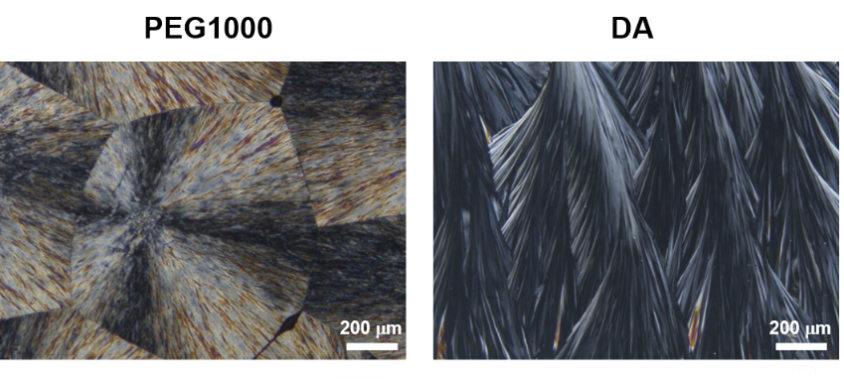
**

**Figure S9.** Polarizing microscope images of PEG1000 and DA in crystalline form.

1. **Full spectra of variable temperature XRD**


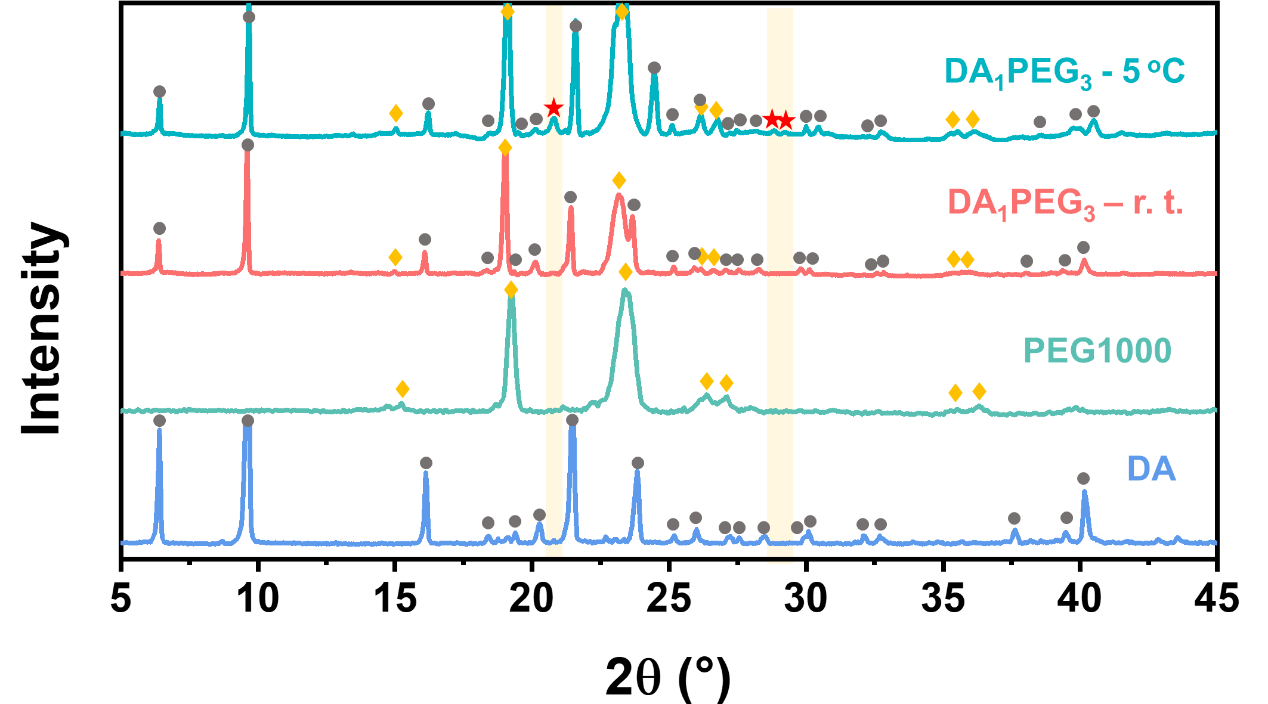


**Figure S10.** XRD data of DA_1_PEG_3_ at r. t. and 5 ^o^C, DA and PEG1000 (•, ♦, ★ represent the diffraction peaks of DA, PEG1000, and the new peaks, respectively).


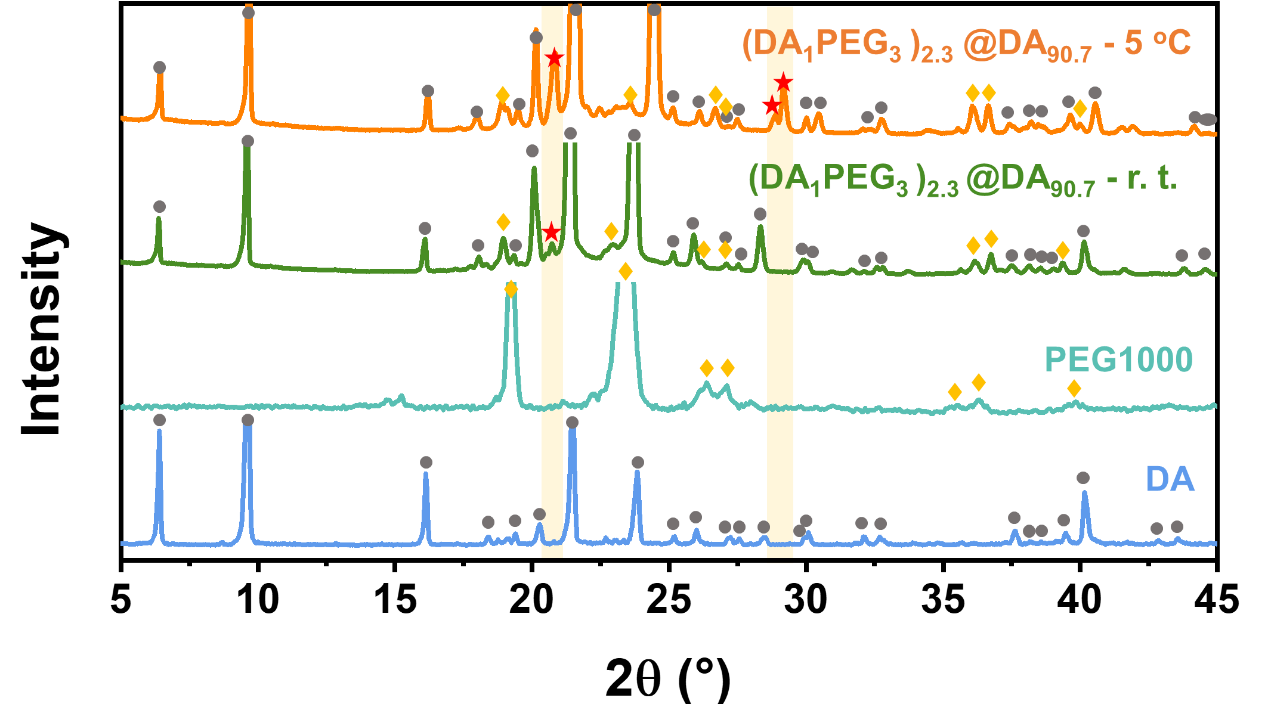


**Figure S11.** XRD data of (DA_1_PEG_3_)_2.3_@DA_90.7_ at r. t. and 5 ^o^C, DA and PEG1000 (•, ♦, ★ represent the diffraction peaks of DA, PEG1000, and the new peaks, respectively).

1. **Effect of changing crystallization point modulator and host matrix on the response temperature of thermofluorochromic materials**

**Table S2.** Melting point (Tm), crystallization point (T_C_) of PEG with different molecular weights.

|  | PEG1500 | PEG2000 | PEG20000 |
| --- | --- | --- | --- |
| Tm (^o^C) | 48.3 | 56.7 | 66.2 |
| T_C_ (^o^C) | 20.0 | 34.1 | 35.8 |

**
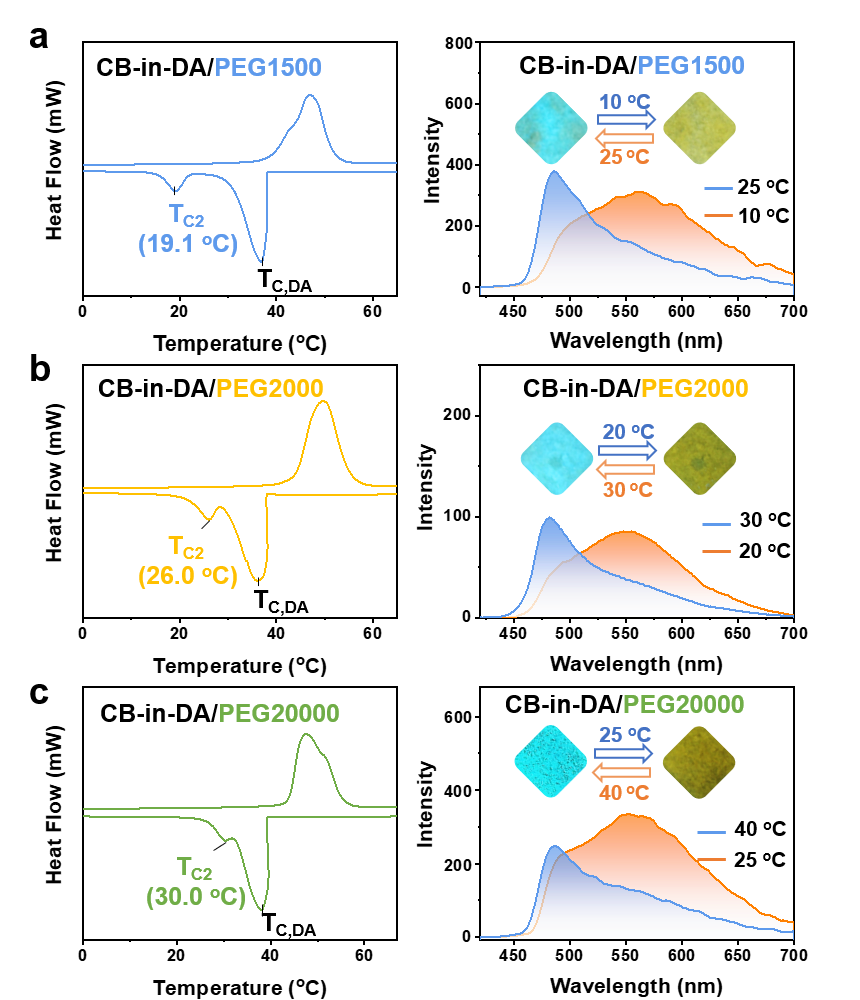
**

**Figure S12.** DSC thermograms, fluorescence spectra and images of (a) CB-in-DA/PEG1500 (*ω*_PEG1500_ = 7 wt%, *C*_CB_ = 1 mg/g), (b) CB-in-DA/PEG2000 (*ω*_PEG2000_ = 7 wt%, *C*_CB_ = 1 mg/g), (c) CB-in-DA/PEG20000 (*ω*_PEG20000_ = 7 wt%, *C*_CB_ = 1 mg/g).

By selecting PEG with different T_C_ (such as PEG1500, PEG2000, and PEG20000) as crystallization point modulators, thermofluorochromic materials with distinct response temperatures can be developed. Specifically: For CB-in-DA/PEG1500, the fluorescence response temperature (T_C2_) is 19.1 ^o^C. When the temperature rises from below the response temperature (e.g. 10 °C) to above the response temperature (e.g. 25 °C), the fluorescence changes from cyan to yellow. The fluorescence response temperature (T_C2_) for CB-in-DA/PEG2000 increases to 26.0 °C, and fluorescence change from cyan to yellow was also observed when switching the temperature between 20 °C and 30 °C. In the case of CB-in-DA/PEG20000, the fluorescence response temperature (T_C2_) further increases to 30.0 °C. As the temperature rises from below the response temperature (e.g. 25 °C) to above the response temperature (e.g. 40 °C), the fluorescence also changes from cyan to yellow.

**Table S3.** Melting point (Tm), crystallization point (T_C_) of TA and HcA.

|  | TA | HcA |
| --- | --- | --- |
| Tm (^o^C) | 56.4 | 64.1 |
| T_C_ (^o^C) | 50.4 | 59.0 |


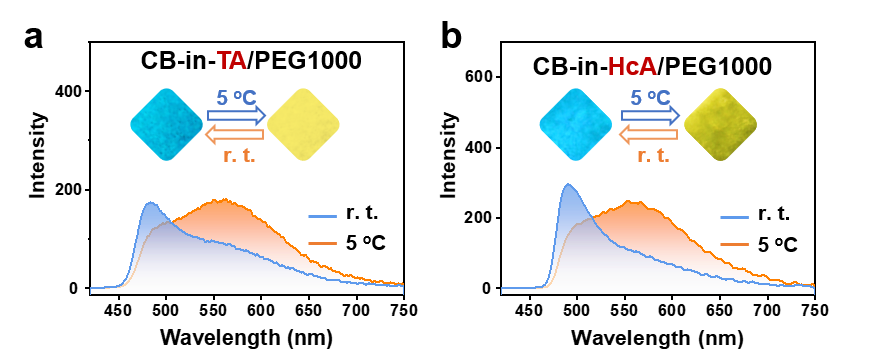


**Figure S13.** Fluorescence spectra and images of (a) CB-in-TA/PEG1000 (*ω*_PEG1000_ = 7 wt%, *C*_CB_ = 1 mg/g), (b) CB-in-HcA/PEG1000 (*ω*_PEG1000_ = 7 wt%, *C*_CB_ = 1 mg/g).

1. **Supplementary Reference**

[S1] K. Huang, M. Liu, X. Wang, D. Cao, F. Gao, K. Zhou, W. Wang, W. Zeng, *Tetrahedron Lett.* **2015**, 56, 3769.

[S2] J. Du, L. Sheng, Y. Xu, Q. Chen, C. Gu, M. Li, S. X.-A. Zhang, *Adv. Mater.* **2021**, 33, 2008055.

[S3] J. Du, L. Sheng, Q. Chen, Y. Xu, W. Li, X. Wang, M. Li, S. X.-A. Zhang, *Mater. Horiz.* **2019**, 6, 1654.
